# Neural and non‐neural contributions to sexual dimorphism of mid‐day sleep in Drosophila melanogaster: a pilot study

**DOI:** 10.1111/phen.12134

**Published:** 2016-02-19

**Authors:** Mobina Khericha, Jaison B. Kolenchery, Eran Tauber

**Affiliations:** ^1^ Department of GeneticsUniversity of LeicesterLeicesterU.K.

**Keywords:** Drosophila, fat body, mushroom body, sexual dimorphism, sleep, takeout, transformer

## Abstract

Many of the characteristics associated with mammalian sleep are also observed in Drosophila melanogaster Meigen, making the fruit fly a powerful model organism for studying the genetics of this important process. Included among the similarities is the presence of sexual dimorphic sleep patterns, which, in flies, are manifested as increased mid‐day sleep (‘siesta’) in males compared with females. In the present study, targeted mis‐expression of the genes transformer (tra) and tra2 is used to either feminize or masculinize specific neural and non‐neural tissues in the fly. Feminization of male D. melanogaster using three different GAL4 drivers that are expressed in the mushroom bodies induces a female‐like reduced siesta, whereas the masculinization of females using these drivers triggers the male‐like increased siesta. A similar reversal of sex‐specific sleep is also observed by mis‐expressing tra in the fat body, which is a key tissue in energy metabolism and hormone secretion. In addition, the daily expression levels of takeout, an important circadian clock output gene, are sexually dimorphic. Taken together, these experiments suggest that sleep sexual dimorphism in D. melanogaster is driven by multiple neural and non‐neural circuits, within and outside the brain.

## Introduction

Studies in a variety of organisms have shown that various sleep properties are sex‐specific. In humans, for example, the frequency of sleep spindles (a burst of oscillatory neural activity during stage N2 sleep) is elevated in women compared with men (Gaillard & Blois, 1981). In addition, women sleep longer, when deprived of external cues under laboratory conditions (Wever, 1984) and slow wave sleep is more frequent in women than in men (Reynolds *et al*., 1990). A sex difference in sleep patterns is also present in mice (Sinton *et al*., 1981; Paul *et al*., 2006) and rats (Fang & Fishbein, 1996).

Similar to mammals, the pattern of sleep in *Drosophila melanogaster* Meigen is also sexually dimorphic, with a pronounced mid‐day sleep (‘siesta’) in males but not in females (Andretic & Shaw, 2005; Ho & Sehgal, 2005). In addition, the response of the fly to sleep deprivation is also reported (Shaw *et al*., 2002; Hendricks *et al*., 2003), although sex dimorphic differences are observed only in the circadian clock mutant cycle (aka *Bmal1*). Female mutants have a pronounced rest rebound, whereas, in males, the homeostatic response is reduced or non‐existing.

A recent study (Catterson *et al*., 2010) reports that diet has a major impact on sleep patterns, in a way that is also sex‐dependent. Males fed with dietary yeast extracts show increased locomotor activity and shortened diurnal and nocturnal sleep, whereas females respond to this diet with reduced daytime locomotor activity and a more fragmented nocturnal sleep. The reduced mid‐day sleep in females is associated mainly with inseminated females (Isaac *et al*., 2010), which suggests that the sex peptide, a male seminal peptide transferred during copulation, modulates female behaviour and promotes their mid‐day waking.

Sex determination in *D. melanogaster* is studied extensively (Schutt & Nothiger, 2000) and genetic tools are available that allow manipulation of specific target tissues. The *transformer* (*tra*) gene is a key gene in the cascade responsible for somatic sexual differentiation. In females, splicing of *tra* (mediated by SXL) generates TRA protein, which activates the female sexual differentiation. In males, the *tra* pre‐mRNA is spliced into its male‐specific form, which translates into a truncated inactive protein, consequently leading to male sexual differentiation. Ectopic expression of the female form of *tra* RNA causes chromosomal males to develop as females (McKeown *et al*., 1988). The UAS‐GAL4 binary system in *Drosophila* (Brand & Perrimon, 1993) allows the expression of the female spliced form of *tra* in targeted cells in a male, inducing a female pattern of development; strains with a GAL4 transgene expressed in a defined set of cells are crossed with those carrying the female‐specific *tra^F^* fused to upstream activating sequence (UAS‐*tra*). This leads to activation of *tra* in all the tissues expressing GAL4, creating tissue‐specific feminization (Ferveur *et al*., 1995, 1997). A similar approach is also used to masculinize female specific tissues using a *tra*2 RNA interfering construct (UAS‐*tra2*‐IR) (Lazareva *et al*., 2007). In the present study, the UAS‐GAL4 system is used to feminize male specific regions of the brain and to masculinize female specific neurones, with the aim of identifying the sleep circuits that control this sexually dimorphic behaviour in flies.

## Materials and methods

### Fly strains

To feminize males, the strain *w*; UAS‐*tra^F^* from the Bloomington *Drosophila* Stock Center at Indiana University (stock number 4590) was used. For female masculinization, a transgenic strain carrying dsRNAi construct targeting *tra2* (UAS‐*tra2*‐IR) was used, which was obtained from the Vienna *Drosophila* RNAi Centre (stock v8868). Another strain targeting UAS‐*tra* has also been used (stock v2560), although preliminary tests indicated that mid‐day sleep in UAS‐*tra*‐IR females is unusually high, and therefore not useful for testing female masculinization. UAS‐*dicer2* transgenic strain (stock v60008) was used to enhance the efficiency of RNAi in some crosses (specified when used).

Four GAL4 enhancer‐trap strains, 103Y, 30Y, 121Y (Gatti *et al*., 2000) and Voila‐GAL4 (Balakireva *et al*., 1998), driving expression in the mushroom bodies, central complex and a small cluster in pars intercerebralis, were a gift from Jean‐François Ferveur at the University of Dijon, France. Additional GAL4 strains obtained from Bloomington Stock Center included the pan neural *w;elav*‐GAL4 (stock 8760) and *w*;1471‐GAL4 strain with expression patterns in the γ lobes of mushroom bodies (stock 9465). *takeout* (*to*)‐GAL4 driving expression in the fat body, as well as in a subset of cells within the maxillary palps and antennae (Dauwalder *et al*., 2002), was a gift from Brigitte Dauwalder at the University of Houston, Texas.

Each of the strains above was also crossed with *w^1118^* and their F1 progeny were used as two controls (UAS and GAL4) compared with the phenotype of flies carrying both transgenes. All stocks and experimental crosses were maintained under an LD 12 : 12 h photocycle at 25 °C and maintained on standard cornmeal/sugar–based food.

### Sleep assay

The sleep/wake pattern of flies aged 3–4 days was monitored using the *Drosophila* Activity Monitoring System (DAMS, TriKinetics, Waltham, Massachusetts) under an LD 12 : 12 h photocycle at 25 °C for a total of 4 days. Only virgin females were used in all experiments. Data were collected in 5‐min bins, and sleep was quantified by summing consecutive bins for which no activity was recorded, using r (R Development Core Team, 2010). Because the mid‐day ‘Siesta’ sleep time interval varied among strains (typically, between 5 and 8 h after lights on), the mid‐day average sleep was quantified during 2 h around noon (5–7 h after lights on). This has simplified the algorithm and ensures the capture of mid‐day sleep. In the feminizing experiments, where the female‐spliced form of *tra* was expressed in males, siesta sleep was calculated in both feminized males and females, and compared with their background controls. Similarly, in the masculinization of females, RNAi constructs of *tra* and *tra*2 were expressed in females, and siesta sleep was assessed in males and masculinized females, and compared with their background controls. In each experiment, the sleep scores of the three genotypes were compared by Kruskal–Wallis analysis of variance. Tests indicating significant difference were followed by the Siegel–Castellan nonparametric post‐hoc test (Siegel & Castellan, 1988), comparing each of the controls with the GAL4/UAS genotype. Statistical tests were carried with the *pgirmess* library implemented in r (R Development Core Team, 2010).

### RNA quantification

The mRNA levels of *to* were assayed by a quantitative polymerase chain reaction (qPCR). Males, virgin females and mated females, aged 4–5 days old, were analyzed. Flies were maintained under an LD 12 : 12 h photocycle at 25 °C for 5 days. On the sixth day, the files were collected at two different time points, immediately after lights on (Zt0) and 6 h after lights off (Zt6). Total RNA was isolated from male fly heads using Trizol (Invitrogen, Carlsbad, California). In total, 500 ng of RNA was used for cDNA synthesis, which was carried with the Affinity Script kit (Stratagene, San Diego, California). Oligo(dT) primers were used for the first‐strand synthesis. qPCR was carried using a SYBR Green assay (Agilent Technologies, Santa Clara, California). The standard curve method was used to quantify *to* mRNA, in 25‐μL reactions, with a final primer concentration of 0.3 µm. The forward primer was, 5′‐GCCTTTTGGTCTCGGTGGAT‐3′; reverse primer, 5′‐TCCCCATTCTTCACCAGCG (amplicon size 142 bp). *Ribosomal protein 49* mRNA (*rp49*) was used as the reference gene. The forward primer was, 5′‐TTACAAGGAGACGGCCAAAG; reverse primer, 5′‐CTCTGCCCACTTGAAGAGC.

## Results

All of the transgenic strains used in the present study exhibited a marked sexually dimorphic mid‐day sleep (see Supporting information, Figures S1–5), with males sleeping for up to twice as much as females (males, mean ± SD: 94 ± 21, females: 42 ± 24 min during 2 h at mid‐day), which is similar to the previously reported sleep differences exhibited by wild‐type Canton‐S (Andretic & Shaw, 2005; Ho & Sehgal, 2005).

**Figure 1:**
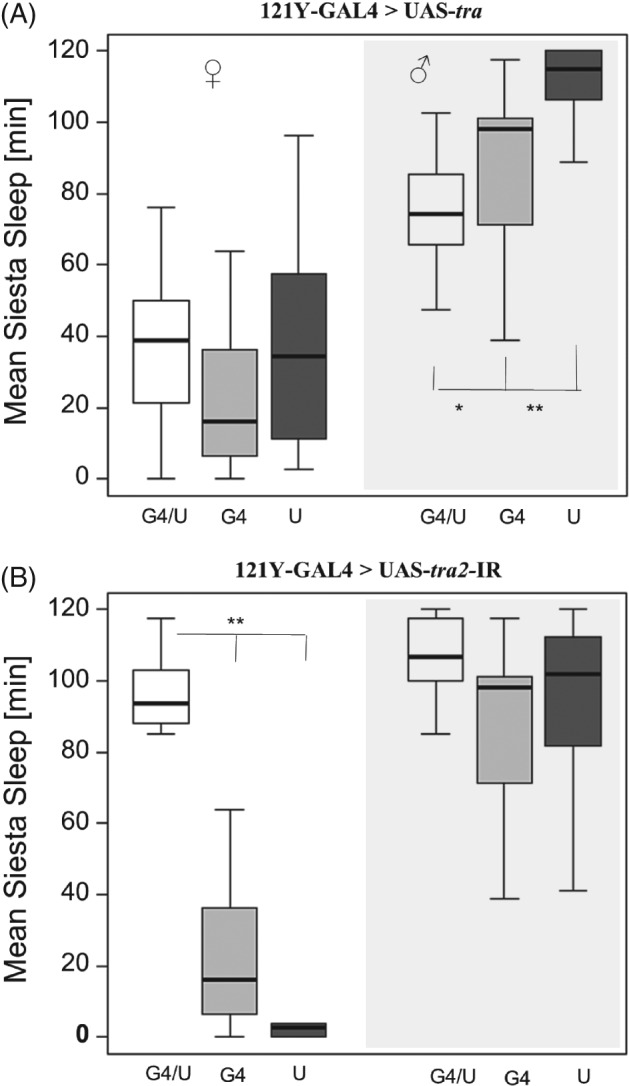
Siesta sleep after feminization and masculinization of mushroom bodies in Drosophila melanogaster. Box plots showing siesta sleep in flies carrying the 121Y‐GAL4 transgene driving (A) UAS‐tra (feminization of males) and (B) UAS–tra2 (masculinization of females). In each panel, the three boxes on the left show sleep in females and the three boxes on the right (shaded grey) show sleep in males. The data represent siesta sleep for the GAL4/UAS genotypes (G4/U, white, n ≥ 20 for all GAL4 lines; males and females) and the single transgene control genotypes (GAL4/+, G4, light grey; UAS/+, U dark grey) for both sexes. Asterisks represent experimental genotype (GAL4/UAS) significance levels compared with control genotypes (GAL4/+ and UAS/+). Nonparametric post‐hoc tests were performed (*P < 0.05, **P < 0.01). The line within each box represents the median siesta sleep (min) averaged over 4 days, and the boxes extend to 25 and 75 percentiles. Note that significance differences are only tested for males in the feminization experiments or females in the masculinization experiments.

In the present study, the contribution of the mushroom bodies to sexual dimorphic sleep in *D. melanogaster* was tested using five different GAL4 drivers. The 121Y‐GAL4 strain drives expression in the central complex and the mushroom bodies (Armstrong *et al*., 1995; Gatti *et al*., 2000). Using this driver to express UAS‐*tra* (Fig. 1A) resulted in significantly reduced (feminized) male siesta sleep compared with control males carrying only a single transgene. Using this driver to knock down *tra2* for masculinization of the mushroom bodies induced siesta sleep in females, which was significantly higher than either of the single transgene controls (Fig. 1B). Note that the similar sleep levels for females in the feminization experiment (Fig. 1A) or males in the masculinization experiment (Fig. 1B) suggest that the observed response is not merely a result of the interaction between the GAL4 and UAS genetic backgrounds.

**Figure 2:**
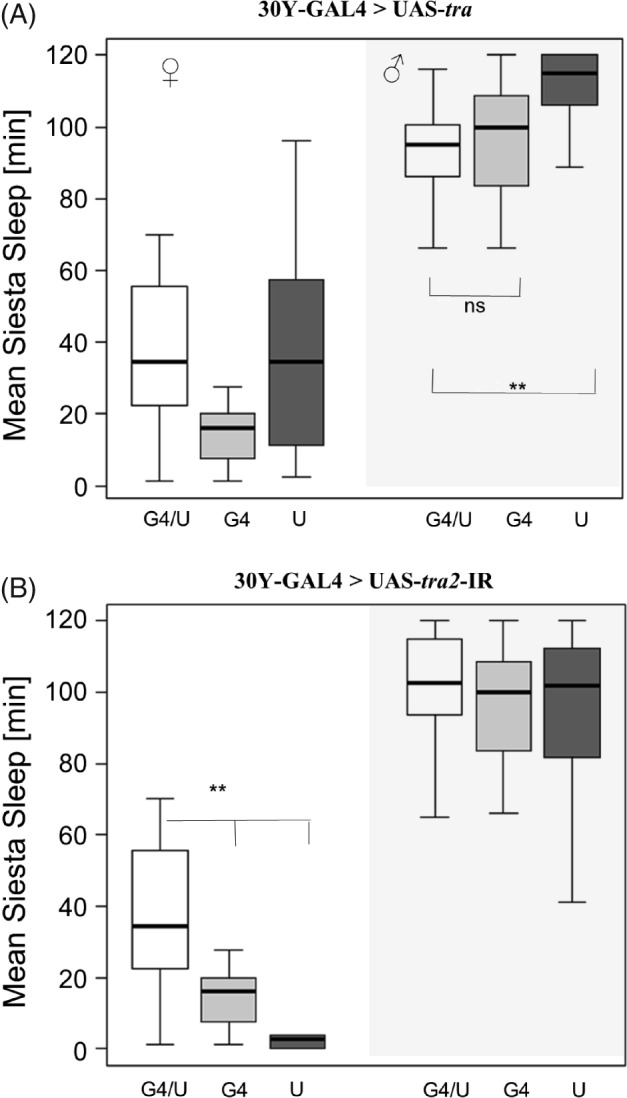
Feminization and masculinization of mushroom bodies in Drosophila melanogaster using a 30Y‐GAL4 transgene. Box plots showing siesta sleep in flies carrying 30Y‐GAL4 driving (A) UAS‐tra (feminization of males) and (B) UAS‐tra2 (masculinization of females). The plotting scheme is the same as in Fig. 1.

The 30Y‐GAL4 transgene is expressed in the mushroom bodies and the central complex (Yang *et al*., 1995; Gatti *et al*., 2000). Feminization of males using this driver induced a small but significant, reduction of sleep compared with the UAS control (*P* < 0.01) but not compared with the GAL4 control, which showed unusual reduced sleep (Fig. 2A). The effect of using this driver for masculinizing females was stronger, and UAS‐*tra2*‐IR (Fig. 2B) resulted in siesta sleep in females comparable with that exhibited by males.

**Figure 3:**
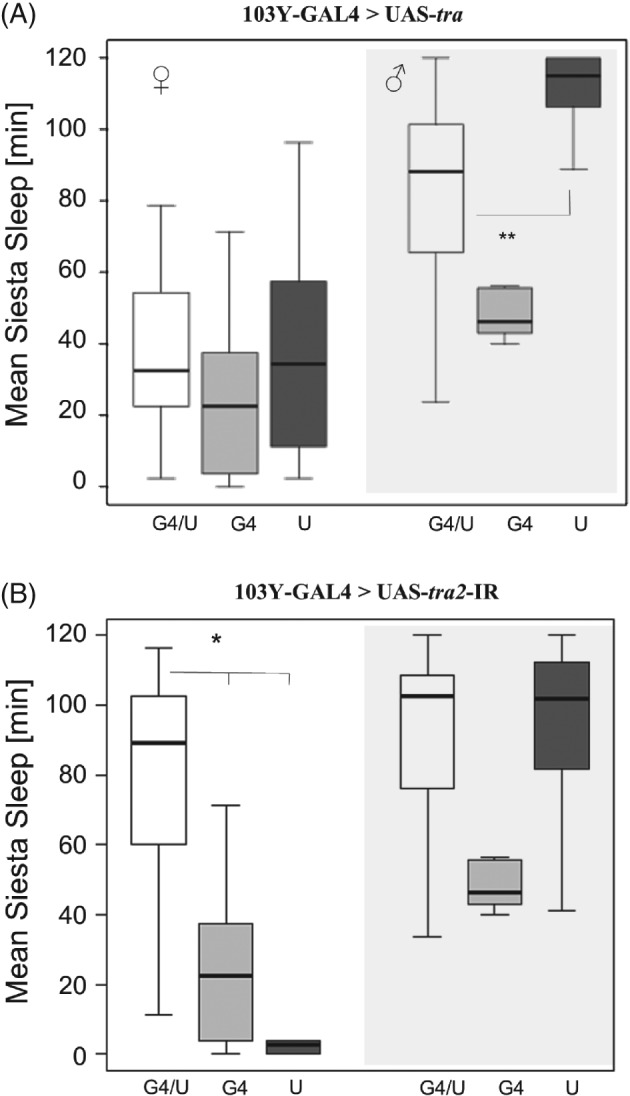
Feminization and masculinization of mushroom bodies in Drosophila melanogaster using a 103Y‐GAL4 transgene. Box plots showing siesta sleep in flies carrying 103Y‐GAL4 driving (A) UAS‐tra (feminization of males) and (B) UAS‐tra2 (masculinization of females). The plotting scheme is the same as in Fig. 1.

**Figure 4:**
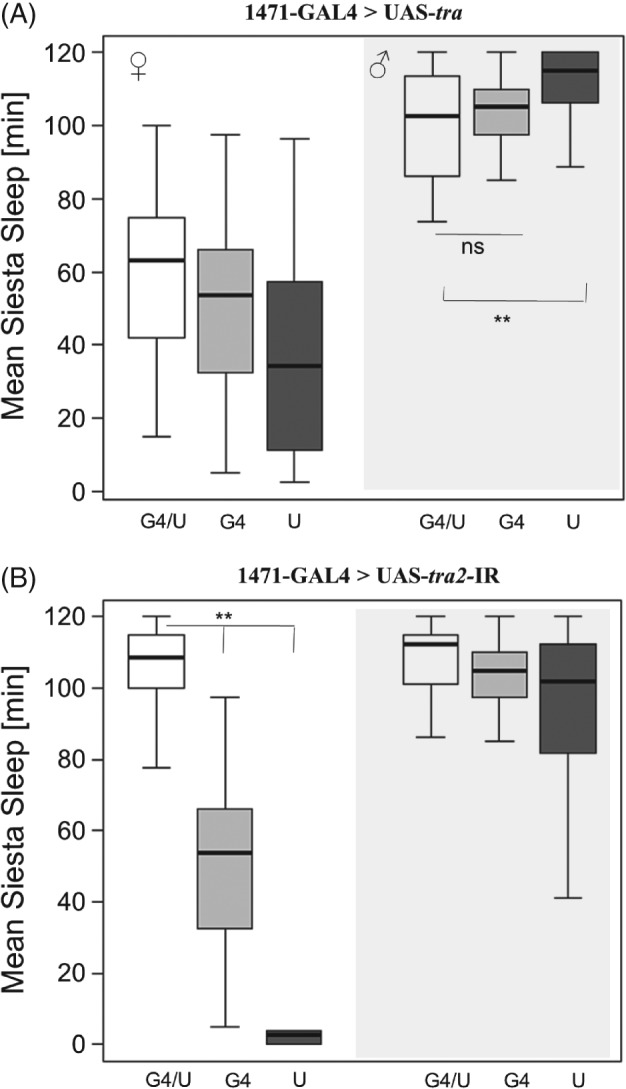
Feminization and masculinization of mushroom bodies in Drosophila melanogaster using a 1471‐GAL4 transgene. Box plots showing siesta sleep in flies carrying 1471‐GAL4 driving (A) UAS‐tra (feminization of males) and (B) UAS‐tra2 (masculinization of females). The plotting scheme is the same as in Fig. 1.

Using the 103Y‐GAL4 line for which expression also extends to the mushroom bodies and central complex (Tettamanti *et al*., 1997) also induced a reversal of siesta sleep; in males, sleep was reduced compared with the UAS control (but not compared with the GAL4 control, which showed nontypical low siesta) (Fig. 3A). In females, brain masculinization induced male‐like siesta sleep (Fig. 3B). A similar reversal of sleep was observed using the 1471‐GAL4, which is expressed in the γ lobes of mushroom bodies (Isabel *et al*., 2004) (Fig. 4). By contrast, use of the *Voila*‐GAL4 line, which is expressed in the mushroom bodies and the antennal lobes (Balakireva *et al*., 1998), did not result in any significant change in sleep in either feminized males or masculinized females (see Supporting information, Figure S6).

**Figure 5:**
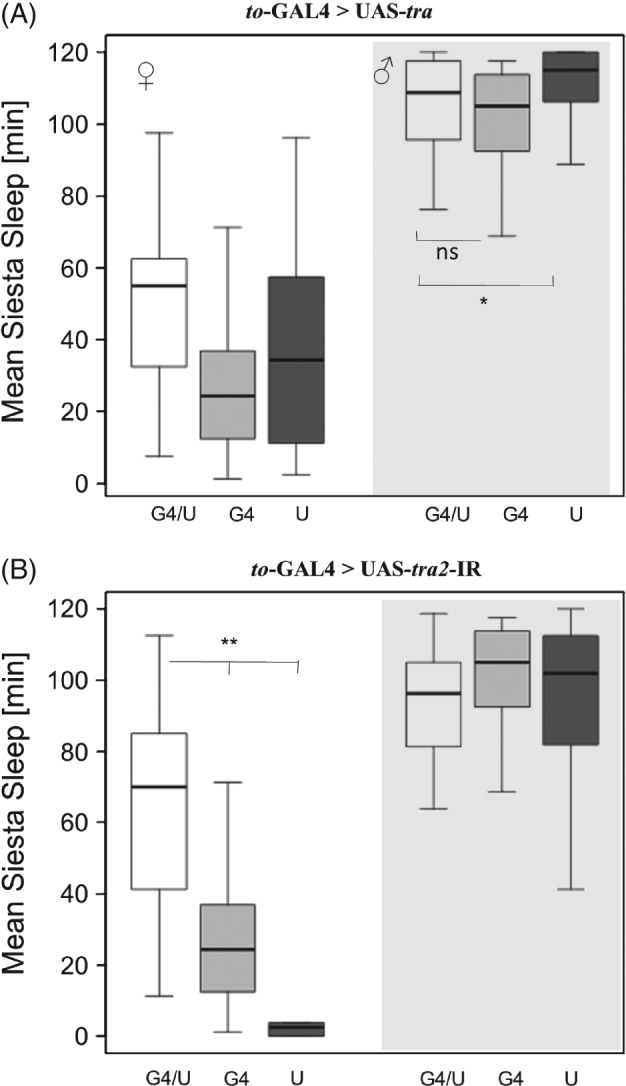
Siesta sleep after feminization and masculinization of the fat body in Drosophila melanogaster. The takeout (to) Gal4 driver was used for (A) feminization of males using UAS‐tra^F^ and (B) masculinization of females using UAS‐tra2‐IR. Plot parameters are as described in Fig. 1.

Interestingly, the *to*‐GAL4 strain, which is expressed in the fat body (Dauwalder *et al*., 2002), was also effective in reversing sleep (Fig. 5). Although feminization of males caused only small reduction of siesta sleep (compared with the UAS but not with the GAL4 control), the masculinization of females using the UAS‐*tra2*‐IR transgene induced a substantial increase in siesta sleep in females (Fig. 5), indicating a role for the fat body in sleep sexual dimorphism.

**Figure 6:**
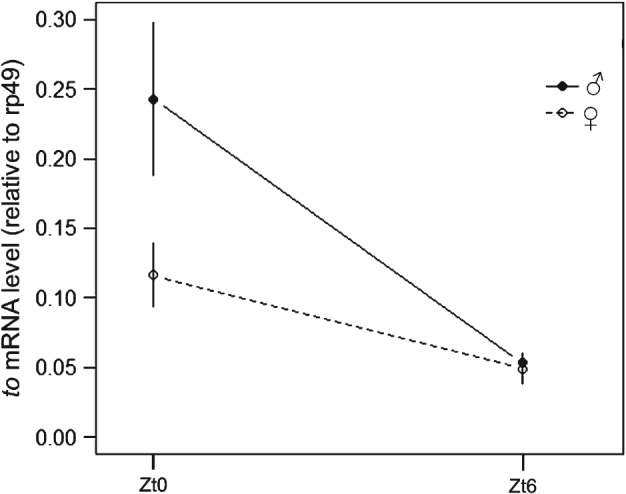
Sexual dimorphism in takeout expression in Drosophila melanogaster. The relative mRNA expression of males (filled circles) and females (open circles) is depicted for Zt0 and Zt6. Expression is normalized to reference gene rp49. The error bars indicate the SE.

The present study also analyzed the transcript level of *to* during the beginning of the day (Zt0) and at midday (Zt6) (Fig. 6). The expression of *to* was sexually dimorphic with a significant time–sex interaction (*F*_1,10_ = 4.99, *P* < 0.05). In both males and females, the transcript level was relatively high at the beginning of the day and declined at midday, as reported previously (Benito *et al*., 2010), although it was substantially higher in males at Zt0 (Fig. 6). Thus, sex‐dependent differences in *to* expression at the beginning of the day may contribute to differences in siesta sleep. Although the RNA level converged to the same level at midday in males and females, there might be a time‐lag between the mRNA and the protein profiles. This would lead to a different level of TO protein between males and females just before siesta time (although previous studies have suggested that this lag is rather small; So *et al*., 2000; Benito *et al*., 2010).

## Discussion

The present study focuses on the mushroom bodies, which are implicated as a key brain structure regarding sleep regulation in the fruit fly *D. melanogaster* (Joiner *et al*., 2006; Pitman *et al*., 2006). The role of the mushroom bodies appears to be complex: preventing mushroom body output (either transiently or by ablation) results in reduced sleep (Joiner *et al*., 2006; Pitman *et al*., 2006), whereas raising the activity of Go signalling in the mushroom body enhances sleep (Guo *et al*., 2011). This complexity is evident from the results of a recent study showing that Go signalling is present in two adjacent subtypes of mushroom body cholinergic neurones with opposite roles in sleep regulation (Yi *et al*., 2013). Most parts of the mushroom body are innervated by a single pair of neurones, the dorsal paired medial (DPM), which is reported to promote sleep (Haynes *et al*., 2015). The mechanism involves inhibition of the mushroom body α′/β′ neurones by GABA release. The mushroom body outputs converge onto a small subset of neurones (called mushroom body output neurones, MBONs), whose role in sleep regulation is reported in detail (Aso *et al*., 2014b). Glutamatergic MBONs are found to be sleep‐suppressing, whereas GABAergic or cholinergic neurones are sleep‐promoting.

Four of the driver lines tested in the present study, 121Y, 30Y, 103Y and Voila, are reported to be implicated in controlling sexually dimorphic locomotion behaviour (Gatti *et al*., 2000), with males exhibiting significantly shorter inter‐bout intervals (and lower variation) than females. The overlap of the expression patterns of these GAL4 lines is restricted to a small cluster in the pars intercerbralis, which is therefore suggested as a candidate for the location of that circuit. In the present study, however, the Voila driver does not have any effect on reversing sleep, whereas the driver *1471‐*GAL4 (not expressed in the pars intercebralis) does (Fig. 4). Given that the overlap between these driver lines consists mainly of the mushroom bodies, as recently shown to be implicated in the regulation of sleep (Joiner *et al*., 2006; Pitman *et al*., 2006), it is likely that neurones in this centre also underlie the variations in siesta sleep. However, it is noted that, in three of the feminization experiments (Figs 2A, 3A and 4A), the experimental line does not differ significantly from the GAL4 driver, complicating the interpretation. Interestingly, males carrying these MB GAL4 driver show unusual sleep, which may be the result of GAL4 accumulation in brain neurones, as is reported elsewhere (Rezaval *et al*., 2007). Testing additional GAL4 drivers with a more specific expression in the mushroom bodies (e.g. using the recently created split‐GAL4 collection; Aso *et al*., 2014a) will aid the identification the neurones underlying sexual dimorphism. In addition, given that the pars intercerebralis is important for sleep regulation (Foltenyi *et al*., 2007; Crocker *et al*., 2010), further analysis using pars intercerebralis‐specific drivers would help to rule out a role for this brain region in the sexual dimorphism. Future experiments would also benefit from backcrossing all GAL4 and UAS strains onto a uniform genetic background, which is rather important in sleep studies involving genetic screens (Axelrod *et al*., 2015).

The use of GAL4 lines may be combined with the GAL80 enhancer traps to repress GAL4 expression, and to drive feminization or masculinization in a subset of cells of the drivers described in the present study, refining the candidate regions (Suster *et al*., 2004). This approach is reported to be very successful for refining the brain neurones that constitute the circadian clock in *Drosophila* (Stoleru *et al*., 2004).

Interestingly, the *to*‐GAL4 strain, which is expressed in the fat body (Dauwalder *et al*., 2002), is also effective in reversing sleep (Fig. 5). The gene *to* is also sparsely expressed in the antennae but not in a sex‐specific manner (Dauwalder *et al*., 2002), and so this tissue is unlikely to contribute to the sleep sexual dimorphism. Previous studies report that *to* is under circadian control (Benito *et al*., 2010) and is involved in the regulation of feeding, as well as adaptation to starvation (Sarov‐Blat *et al*., 2000; Meunier *et al*., 2007). Thus, it is possible that the sleep sexual dimorphism is mediated by *to* (and the fat body) indirectly, such that feminizing or masculinizing the fat body changes the feeding status of the animal, and consequently its foraging behaviour. This idea fits well the recent studies showing a direct link between sleep pattern and feeding (Catterson *et al*., 2010). Interestingly, a recent study analyzing sleep behaviour in wild populations over a broad latitudinal range (Svetec *et al*., 2015) identified *to* as a strongly differentially expressed gene, suggesting that it is the target for natural selection.

The sexual dimorphism in sleep is also attributed to the egg‐laying activity of females (Isaac *et al*., 2010), which, in flies, is also under circadian‐clock regulation (Sheeba *et al*., 2001). Oviposition by itself, cannot explain the reduced mid‐day sleep because it peaks after dusk (Sheeba *et al*., 2001), although females may need to be active during mid‐day to acquire nutrients for egg production, and these sex‐specific metabolic constraints may underlie the sleep sexual dimorphism. However, in the present study, only young virgin females are used, thus excluding oviposition as a major factor for the lack of siesta in females (as is observed in the present study in all GAL4 and UAS strains, as well as Canton‐S). This is also in apparent contradiction to Isaac *et al*. (2010) who report that virgin females show a male‐like siesta, and switch to mid‐day activity after mating because of the effect of the sex peptides transferred by the males. However, the substantially increased mid‐day arousal in virgin females compared with males that is observed in the present study is also reported by others (Harbison *et al*., 2009). Any discrepancy between the studies may be a result of the different strains used but, in general, other mechanisms in addition to the sex peptides appear to contribute to the decreased mid‐day sleep of females. These mechanisms may include both neural and non‐neural circuits, as is suggested by the results of the present study.

## Supporting information


**Figure S1.** Sleep profiles in flies carrying the to‐GAL4 driver. The average total sleep (# 5 min bins/h) over 4 days is depicted (n ≥ 20 for all genotypes). UAS‐tra (feminization of males) genotype (A) and UAS‐tra2 (masculinization of females) genotype (B) of males and females (M and F, respectively). Error bars indicate the SE.
**Figure S2.** Sleep profiles in flies carrying the 30Y‐GAL4 driver. The average total sleep (# 5 min bins/h) over 4 days is depicted (n ≥ 20 for all genotypes). UAS‐tra (feminization of males) genotype (A) and UAS‐tra2 (masculinization of females) genotype (B) of males and females (M and F, respectively). Error bars indicate the SE.
**Figure S3.** Sleep profiles in flies carrying the 1471‐GAL4 driver. The average total sleep (# 5 min bins/h) over 4 days is depicted (n ≥ 20 for all genotypes). UAS‐tra (feminization of males) genotype (A) and UAS‐tra2 (masculinization of females) genotype (B) of males and females (M and F, respectively). Error bars indicate the SE.
**Figure S4.** Sleep profiles in flies carrying the 103Y‐GAL4 driver. The average total sleep (# 5 min bins/h) over 4 days is depicted (n ≥ 20 for all genotypes). UAS‐tra (feminization of males) genotype (A) and UAS‐tra2 (masculinization of females) genotype (B) of males and females (M and F, respectively). Error bars indicate the SE.
**Figure S5.** Sleep profiles in flies carrying the voila‐GAL4 driver. The average total sleep (# 5 min bins/h) over 4 days is depicted (n ≥ 20 for all genotypes). UAS‐tra (feminization of males) genotype (A) and UAS‐tra2 (masculinization of females) genotype (B) of males and females (M and F, respectively). Error bars indicate the SE.
**Figure S6.** Siesta sleep after feminization and masculinization using the Voila‐GAL4 driver. Box plots showing siesta sleep in flies carrying voila‐GAL4 driving (A) UAS‐tra (feminization of males) and (B) UAS‐tra2 (masculinization of females). The three boxes to the left show sleep in females and the three boxes to the right (shaded grey) show sleep in males. The data represent siesta sleep for the GAL4/UAS genotypes (white, n ≥ 20 for all GAL4 lines; males and females) and the single transgene control genotypes (GAL4/+; light grey, UAS/+; dark grey) for both sexes. The line within each box represents the median siesta sleep (min) averaged over 4 days, and the boxes extend to 25 and 75 percentiles. No significant differences were present in males in the feminization experiments, or among the females in the masculinization experiments.

## References

[phen12134-bib-0001] Andretic, R. & Shaw, P.J. (2005) Essentials of sleep recordings in *Drosophila*: moving beyond sleep time. Methods in Enzymology, 393, 759–772. 15817323 10.1016/S0076-6879(05)93040-1

[phen12134-bib-0002] Armstrong, J.D. , Kaiser, K. , Muller, A. *et al* (1995) Flybrain, an on‐line atlas and database of the *Drosophila* nervous system. Neuron, 15, 17–20. 7619521 10.1016/0896-6273(95)90059-4

[phen12134-bib-0003] Aso, Y. , Hattori, D. , Yu, Y. *et al* (2014a) The neuronal architecture of the mushroom body provides a logic for associative learning. Elife, 3, e04577. 25535793 10.7554/eLife.04577 PMC4273437

[phen12134-bib-0004] Aso, Y. , Sitaraman, D. , Ichinose, T. *et al* (2014b) Mushroom body output neurons encode valence and guide memory‐based action selection in *Drosophila* . Elife, 3, e04580. 25535794 10.7554/eLife.04580 PMC4273436

[phen12134-bib-0005] Axelrod, S. , Saez, L. & Young, M.W. (2015) Studying circadian rhythm and sleep using genetic screens in *Drosophila* . Methods in Enzymology, 551, 3–27. 25662449 10.1016/bs.mie.2014.10.026

[phen12134-bib-0006] Balakireva, M. , Stocker, R.F. , Gendre, N. & Ferveur, J.F. (1998) Voila, a new *Drosophila* courtship variant that affects the nervous system: behavioral, neural, and genetic characterization. Journal of Neuroscience, 18, 4335–4343. 9592110 10.1523/JNEUROSCI.18-11-04335.1998 PMC6792809

[phen12134-bib-0007] Benito, J. , Hoxha, V. , Lama, C. *et al* (2010) The circadian output gene *takeout* is regulated by *Pdp1ϵ* . Proceedings of the National Academy of Sciences of the United States of America, 107, 2544–2549. 20133786 10.1073/pnas.0906422107 PMC2823862

[phen12134-bib-0008] Brand, A.H. & Perrimon, N. (1993) Targeted gene expression as a means of altering cell fates and generating dominant phenotypes. Development, 118, 401–415. 8223268 10.1242/dev.118.2.401

[phen12134-bib-0009] Catterson, J.H. , Knowles‐Barley, S. , James, K. *et al* (2010) Dietary modulation of *Drosophila* sleep–wake behaviour. PLoS ONE, 5, e12062. 20706579 10.1371/journal.pone.0012062 PMC2919389

[phen12134-bib-0010] Crocker, A. , Shahidullah, M. , Levitan, I.B. & Sehgal, A. (2010) Identification of a neural circuit that underlies the effects of octopamine on sleep: wake behavior. Neuron, 65, 670–681. 20223202 10.1016/j.neuron.2010.01.032 PMC2862355

[phen12134-bib-0011] Dauwalder, B. , Tsujimoto, S. , Moss, J. & Mattox, W. (2002) The *Drosophila takeout* gene is regulated by the somatic sex‐determination pathway and affects male courtship behavior. Genes & Development, 16, 2879–2892. 12435630 10.1101/gad.1010302 PMC187483

[phen12134-bib-0012] Fang, J. & Fishbein, W. (1996) Sex differences in paradoxical sleep: influences of estrus cycle and ovariectomy. Brain Research, 734, 275–285. 8896835

[phen12134-bib-0013] Ferveur, J.F. , Stortkuhl, K.F. , Stocker, R.F. & Greenspan, R.J. (1995) Genetic feminization of brain structures and changed sexual orientation in male *Drosophila* . Science, 267, 902–905. 7846534 10.1126/science.7846534

[phen12134-bib-0014] Ferveur, J.F. , Savarit, F. , O'Kane, C.J. *et al* (1997) Genetic feminization of pheromones and its behavioral consequences in *Drosophila* males. Science, 276, 1555–1558. 9171057 10.1126/science.276.5318.1555

[phen12134-bib-0015] Foltenyi, K. , Greenspan, R.J. & Newport, J.W. (2007) Activation of EGFR and ERK by rhomboid signaling regulates the consolidation and maintenance of sleep in *Drosophila* . Natare Neuroscience, 10, 1160–1167. 17694052 10.1038/nn1957

[phen12134-bib-0016] Gaillard, J.M. & Blois, R. (1981) Spindle density in sleep of normal subjects. Sleep, 4, 385–391. 7313391 10.1093/sleep/4.4.385

[phen12134-bib-0017] Gatti, S. , Ferveur, J.F. & Martin, J.R. (2000) Genetic identification of neurons controlling a sexually dimorphic behaviour. Current Biology, 10, 667–670. 10837249 10.1016/s0960-9822(00)00517-0

[phen12134-bib-0018] Guo, F. , Yi, W. , Zhou, M. & Guo, A. (2011) Go signaling in mushroom bodies regulates sleep in *Drosophila* . Sleep, 34, 273–281. 21358844 10.1093/sleep/34.3.273 PMC3041703

[phen12134-bib-0019] Harbison, S.T. , Carbone, M.A. , Ayroles, J.F. *et al* (2009) Co‐regulated transcriptional networks contribute to natural genetic variation in *Drosophila* sleep. Nature Genetics, 41, 371–375. 19234472 10.1038/ng.330 PMC2683981

[phen12134-bib-0020] Haynes, P.R. , Christmann, B.L. & Griffith, L.C. (2015) A single pair of neurons links sleep to memory consolidation in *Drosophila melanogaster* . Elife, 4 DOI: 10.7554/eLife.03868. 10.7554/eLife.03868 PMC4305081 25564731

[phen12134-bib-0021] Hendricks, J.C. , Lu, S. , Kume, K. *et al* (2003) Gender dimorphism in the role of cycle (BMAL1) in rest, rest regulation and longevity in *Drosophila melanogaster* . Journal of Biological Rhythms, 18, 12–25. 12568241 10.1177/0748730402239673

[phen12134-bib-0022] Ho, K.S. & Sehgal, A. (2005) *Drosophila melanogaster*: an insect model for fundamental studies of sleep. Methods in Enzymology, 393, 772–793. 15817324 10.1016/S0076-6879(05)93041-3

[phen12134-bib-0023] Isaac, R.E. , Li, C. , Leedale, A.E. & Shirras, A.D. (2010) *Drosophila* male sex peptide inhibits siesta sleep and promotes locomotor activity in the post‐mated female. Proceedings of the Royal Society of London Series B, Biological Sciences, 277, 65–70. 19793753 10.1098/rspb.2009.1236 PMC2842620

[phen12134-bib-0024] Isabel, G. , Pascual, A. & Preat, T. (2004) Exclusive consolidated memory phases in *Drosophila* . Science, 304, 1024–1027. 15143285 10.1126/science.1094932

[phen12134-bib-0025] Joiner, W.J. , Crocker, A. , White, B.H. & Sehgal, A. (2006) Sleep in *Drosophila* is regulated by adult mushroom bodies. Nature, 441, 757–760. 16760980 10.1038/nature04811

[phen12134-bib-0026] Lazareva, A.A. , Roman, G. , Mattox, W. *et al* (2007) A role for the adult fat body in *Drosophila* male courtship behavior. PLoS Genetics, 3, e16. 17257054 10.1371/journal.pgen.0030016 PMC1781494

[phen12134-bib-0027] McKeown, M. , Belote, J.M. & Boggs, R.T. (1988) Ectopic expression of the female transformer gene product leads to female differentiation of chromosomally male *Drosophila* . Cell, 53, 887–895. 2454747 10.1016/s0092-8674(88)90369-8

[phen12134-bib-0028] Meunier, N. , Belgacem, Y.H. & Martin, J.R. (2007) Regulation of feeding behaviour and locomotor activity by takeout in *Drosophila* . Journal of Experimental Biology, 210, 1424–1434. 17401125 10.1242/jeb.02755

[phen12134-bib-0029] Paul, K.N. , Dugovic, C. , Turek, F.W. & Laposky, A.D. (2006) Diurnal sex differences in the sleep–wake cycle of mice are dependent on gonadal function. Sleep, 29, 1211–1223. 17040009 10.1093/sleep/29.9.1211

[phen12134-bib-0030] Pitman, J.L. , McGill, J.J. , Keegan, K.P. & Allada, R. (2006) A dynamic role for the mushroom bodies in promoting sleep in *Drosophila* . Nature, 441, 753–756. 16760979 10.1038/nature04739

[phen12134-bib-0031] R Development Core Team (2010) R: A Language and Environment for Statistical Computing, 2.10.1. R Foundation, Austria.

[phen12134-bib-0032] Reynolds, C.F. III , Kupfer, D.J. , Thase, M.E. *et al* (1990) Sleep, gender, and depression: an analysis of gender effects on the electroencephalographic sleep of 302 depressed outpatients. Biological Psychiatry, 28, 673–684. 2242388 10.1016/0006-3223(90)90454-a

[phen12134-bib-0033] Rezaval, C. , Werbajh, S. & Ceriani, M.F. (2007) Neuronal death in *Drosophila* triggered by GAL4 accumulation. European Journal of Neuroscience, 25, 683–694. 17313569 10.1111/j.1460-9568.2007.05317.x

[phen12134-bib-0034] Sarov‐Blat, L. , So, W.V. , Liu, L. & Rosbash, M. (2000) The *Drosophila takeout* gene is a novel molecular link between circadian rhythms and feeding behavior. Cell, 101, 647–656. 10892651 10.1016/s0092-8674(00)80876-4

[phen12134-bib-0035] Schutt, C. & Nothiger, R. (2000) Structure, function and evolution of sex‐determining systems in Dipteran insects. Development, 127, 667–677. 10648226 10.1242/dev.127.4.667

[phen12134-bib-0036] Shaw, P.J. , Tononi, G. , Greenspan, R.J. & Robinson, D.F. (2002) Stress response genes protect against lethal effects of sleep deprivation in *Drosophila* . Nature, 417, 287–291. 12015603 10.1038/417287a

[phen12134-bib-0037] Sheeba, V. , Chandrashekaran, M.K. , Joshi, A. & Kumar Sharma, V. (2001) A case for multiple oscillators controlling different circadian rhythms in *Drosophila melanogaster* . Journal of Insect Physiology, 47, 1217–1225. 12770200 10.1016/s0022-1910(01)00107-x

[phen12134-bib-0038] Siegel, S. & Castellan, N.J. Jr. (1988) Nonparametric Statistics for the Behavioral Sciences, 2nd edn. McGraw‐Hill, New York, New York.

[phen12134-bib-0039] Sinton, C.M. , Valatx, J.L. & Jouvet, M. (1981) Increased sleep time in the offspring of caffeine‐treated dams from two inbred strains of mice. Neuroscience Letters, 24, 169–174. 7254714 10.1016/0304-3940(81)90243-3

[phen12134-bib-0040] So, W.V. , Sarov‐Blat, L. , Kotarski, C.K. *et al* (2000) takeout, a novel *Drosophila* gene under circadian clock transcriptional regulation. Molecular and Cellular Biology, 20, 6935–6944. 10958689 10.1128/mcb.20.18.6935-6944.2000 PMC88769

[phen12134-bib-0041] Stoleru, D. , Peng, Y. , Agosto, J. & Rosbash, M. (2004) Coupled oscillators control morning and evening locomotor behaviour of *Drosophila* . Nature, 431, 862–868. 15483615 10.1038/nature02926

[phen12134-bib-0042] Suster, M.L. , Seugnet, L. , Bate, M. & Sokolowski, M.B. (2004) Refining GAL4‐driven transgene expression in *Drosophila* with a GAL80 enhancer‐trap. Genesis, 39, 240–245. 15286996 10.1002/gene.20051

[phen12134-bib-0043] Svetec, N. , Zhao, L. , Saelao, P. *et al* (2015) Evidence that natural selection maintains genetic variation for sleep in *Drosophila melanogaster* . BMC Evolutionary Biology, 15, 41. 25887180 10.1186/s12862-015-0316-2 PMC4374177

[phen12134-bib-0044] Tettamanti, M. , Armstrong, J.D. , Endo, K. *et al* (1997) Early development of the *Drosophila* mushroom bodies, brain centres for associative learning and memory. Development Genes and Evolution, 207, 242–252. 27747422 10.1007/s004270050112

[phen12134-bib-0045] Wever, R.A. (1984) Properties of human sleep–wake cycles: parameters of internally synchronized free‐running rhythms. Sleep, 7, 27–51. 6718923 10.1093/sleep/7.1.27

[phen12134-bib-0046] Yang, M.Y. , Armstrong, J.D. , Vilinsky, I. *et al* (1995) Subdivision of the *Drosophila* mushroom bodies by enhancer‐trap expression patterns. Neuron, 15, 45–54. 7619529 10.1016/0896-6273(95)90063-2

[phen12134-bib-0047] Yi, W. , Zhang, Y. , Tian, Y. *et al* (2013) A subset of cholinergic mushroom body neurons requires go signaling to regulate sleep in *Drosophila* . Sleep, 36, 1809–1821. 24293755 10.5665/sleep.3206 PMC3825430

