# Supplementary material for: Neural and non‐neural contributions to sexual dimorphism of mid‐day sleep in Drosophila melanogaster: a pilot study
Source: Physiol Entomol. 2016 Feb 19;41(4):327–34. doi: 10.1111/phen.12134 (PMC5091642; doi:10.1111/phen.12134)
Supplement: Supplementary file 1 — Figure S1. Sleep profiles in flies carrying the to‐GAL4 driver. The average total sleep (# 5 min bins/h) over 4 days is depicted (n ≥ 20 for all genotypes). UAS‐tra (feminization of males) genotype (A) and UAS‐tra2 (masculinization of females) genotype (B) of males and females (M and F, respectively). Error bars indicate the SE. Figure S2. Sleep profiles in flies carrying the 30Y‐GAL4 driver. The average total sleep (# 5 min bins/h) over 4 days is depicted (n ≥ 20 for all genotypes). UAS‐tra (feminization of males) genotype (A) and UAS‐tra2 (masculinization of females) genotype (B) of males and females (M and F, respectively). Error bars indicate the SE. Figure S3. Sleep profiles in flies carrying the 1471‐GAL4 driver. The average total sleep (# 5 min bins/h) over 4 days is depicted (n ≥ 20 for all genotypes). UAS‐tra (feminization of males) genotype (A) and UAS‐tra2 (masculinization of females) genotype (B) of males and females (M and F, respectively). Error bars indicate the SE. Figure S4. Sleep profiles in flies carrying the 103Y‐GAL4 driver. The average total sleep (# 5 min bins/h) over 4 days is depicted (n ≥ 20 for all genotypes). UAS‐tra (feminization of males) genotype (A) and UAS‐tra2 (masculinization of females) genotype (B) of males and females (M and F, respectively). Error bars indicate the SE. Figure S5. Sleep profiles in flies carrying the voila‐GAL4 driver. The average total sleep (# 5 min bins/h) over 4 days is depicted (n ≥ 20 for all genotypes). UAS‐tra (feminization of males) genotype (A) and UAS‐tra2 (masculinization of females) genotype (B) of males and females (M and F, respectively). Error bars indicate the SE. Figure S6. Siesta sleep after feminization and masculinization using the Voila‐GAL4 driver. Box plots showing siesta sleep in flies carrying voila‐GAL4 driving (A) UAS‐tra (feminization of males) and (B) UAS‐tra2 (masculinization of females). The three boxes to the left show sleep in females and the three boxes to the right (shaded gre [file PHEN-41-327-s001.pdf]

Supplementary Material

Neural and non-neural contributions to sexual dimorphism of mid-day sleep in Drosophila: A pilot study

Khericha M, Kolenchery JB, Tauber E*

Dept. Genetics, University of Leicester, Leicester United Kingdom

*** Correspondence:** Dr Eran Tauber Dept. of Genetics. University of Leicester. Leicester LE1 7RH United Kingdom
et22@le.ac.uk

Supplementary Figures (S1 – S6)

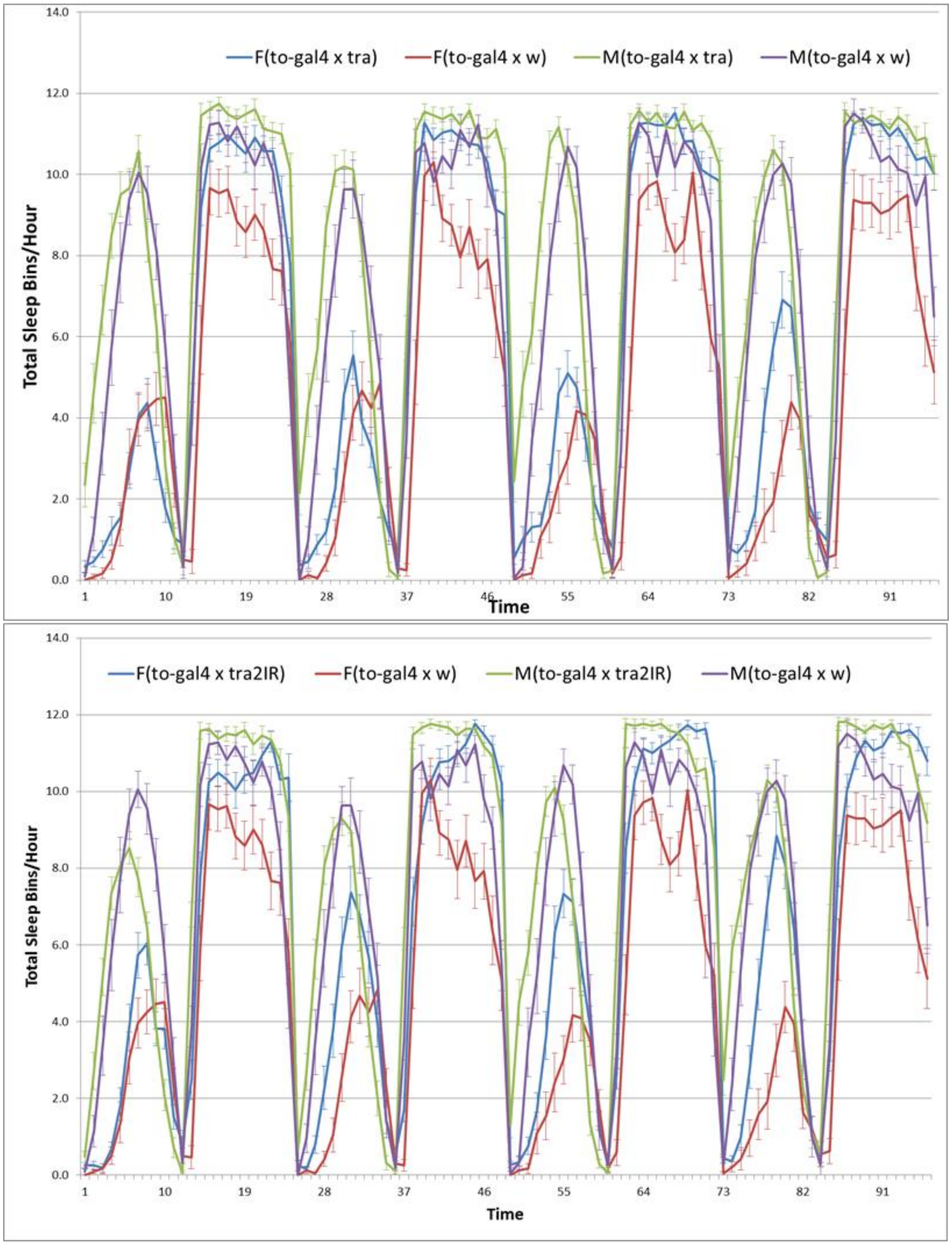

Figure S1. Sleep profiles in flies carrying the *to*-GAL4 driver. The average total sleep (# 5 min bins/hour) over four days is depicted (n= > 20 for all genotypes). UAS-*tra* (feminsation of males) genotype (**top**) and UAS –*tra2* (masculinisation of females) genotype (**bottom**) of males females (M and F respectively). Error bars represent SE.

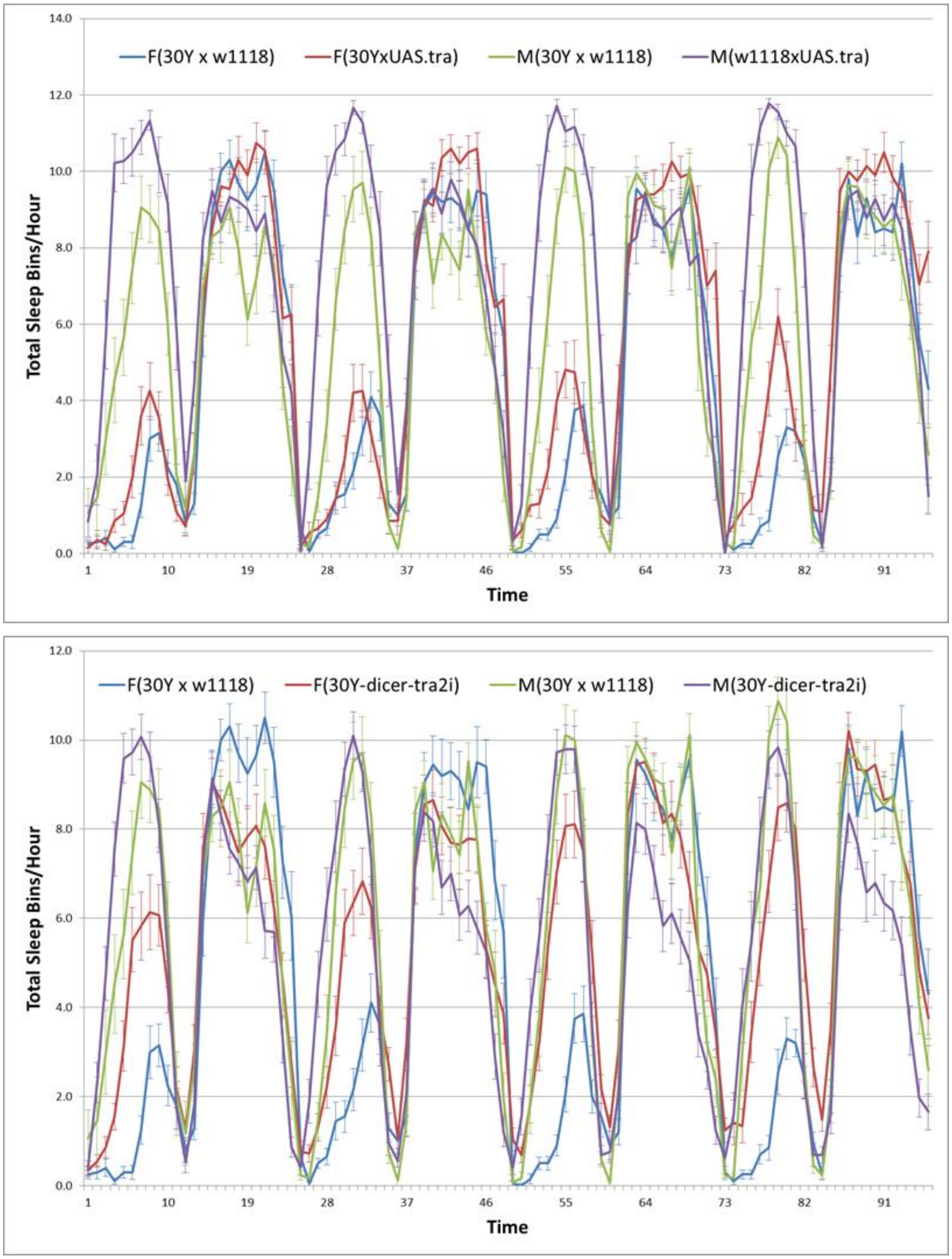

Figure S2. Sleep profiles in flies carrying the 30Y-GAL4 driver. The average total sleep (# 5 min bins/hour) over four days is depicted (n= > 20 for all genotypes). UAS-*tra* (feminsation of males) genotype (**top**) and UAS –*tra2* (masculinisation of females) genotype (**bottom**) of males females (M and F respectively). Error bars represent SE.

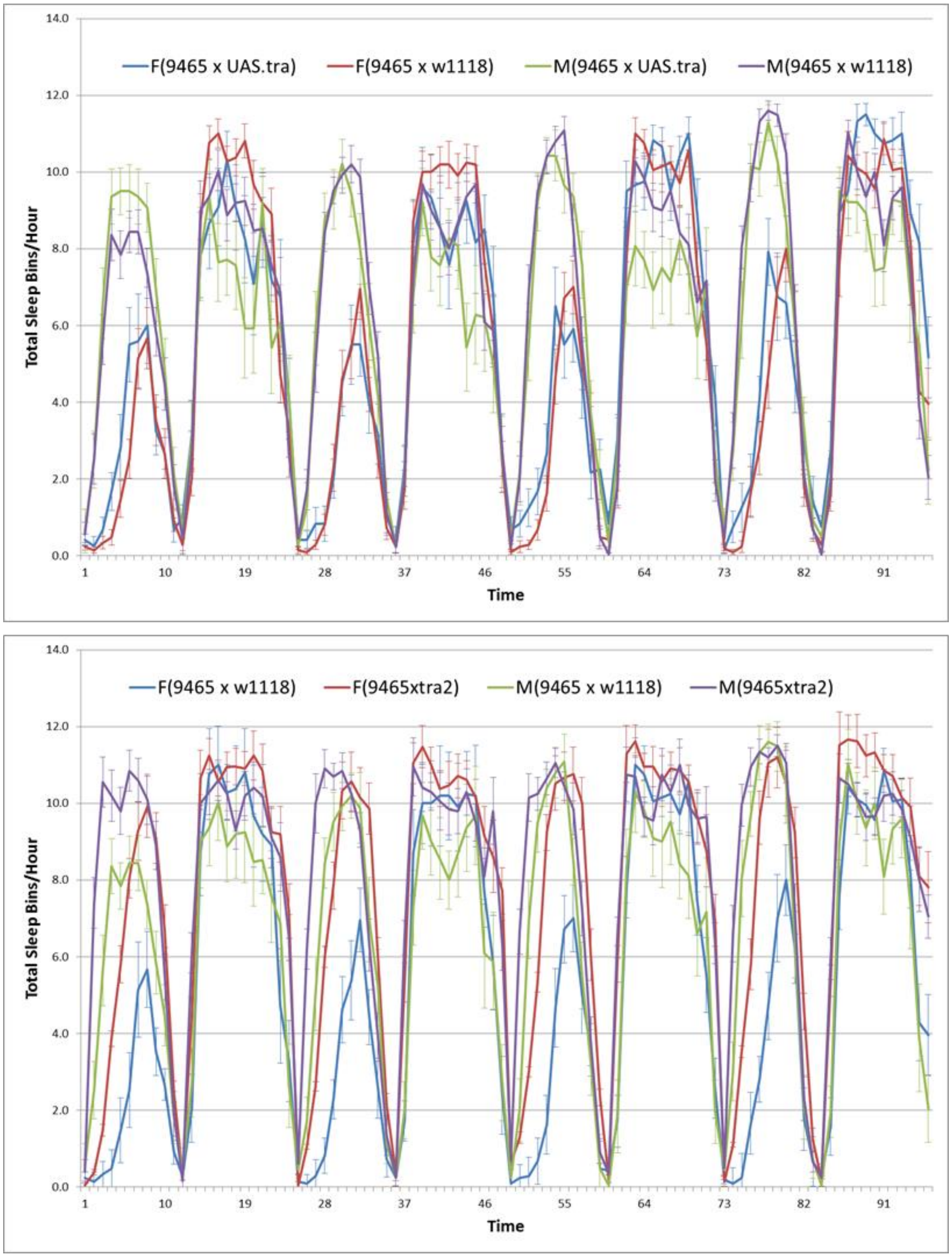

Figure S3. Sleep profiles in flies carrying the 1471-GAL4 driver. The average total sleep (# 5 min bins/hour) over four days is depicted (n= > 20 for all genotypes). UAS-*tra* (feminsation of males) genotype (**top**) and UAS –*tra2* (masculinisation of females) genotype (**bottom**) of males females (M and F respectively). Error bars represent SE.

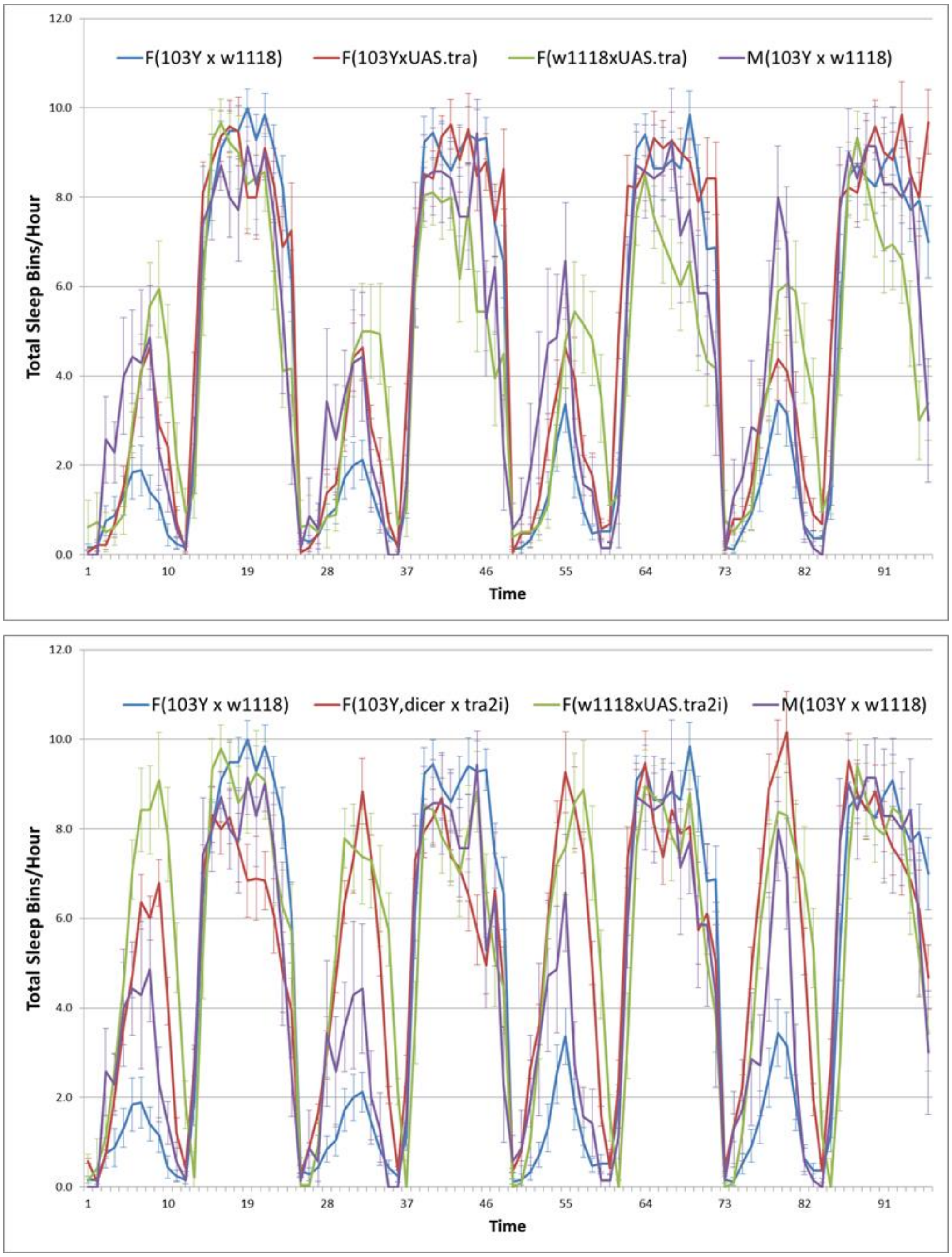

Figure S4. Sleep profiles in flies carrying the 103Y-GAL4 driver. The average total sleep (# 5 min bins/hour) over four days is depicted (n= > 20 for all genotypes). UAS-*tra* (feminsation of males) genotype (**top**) and UAS –*tra2* (masculinisation of females) genotype (**bottom**) of males females (M and F respectively). Error bars represent SE.

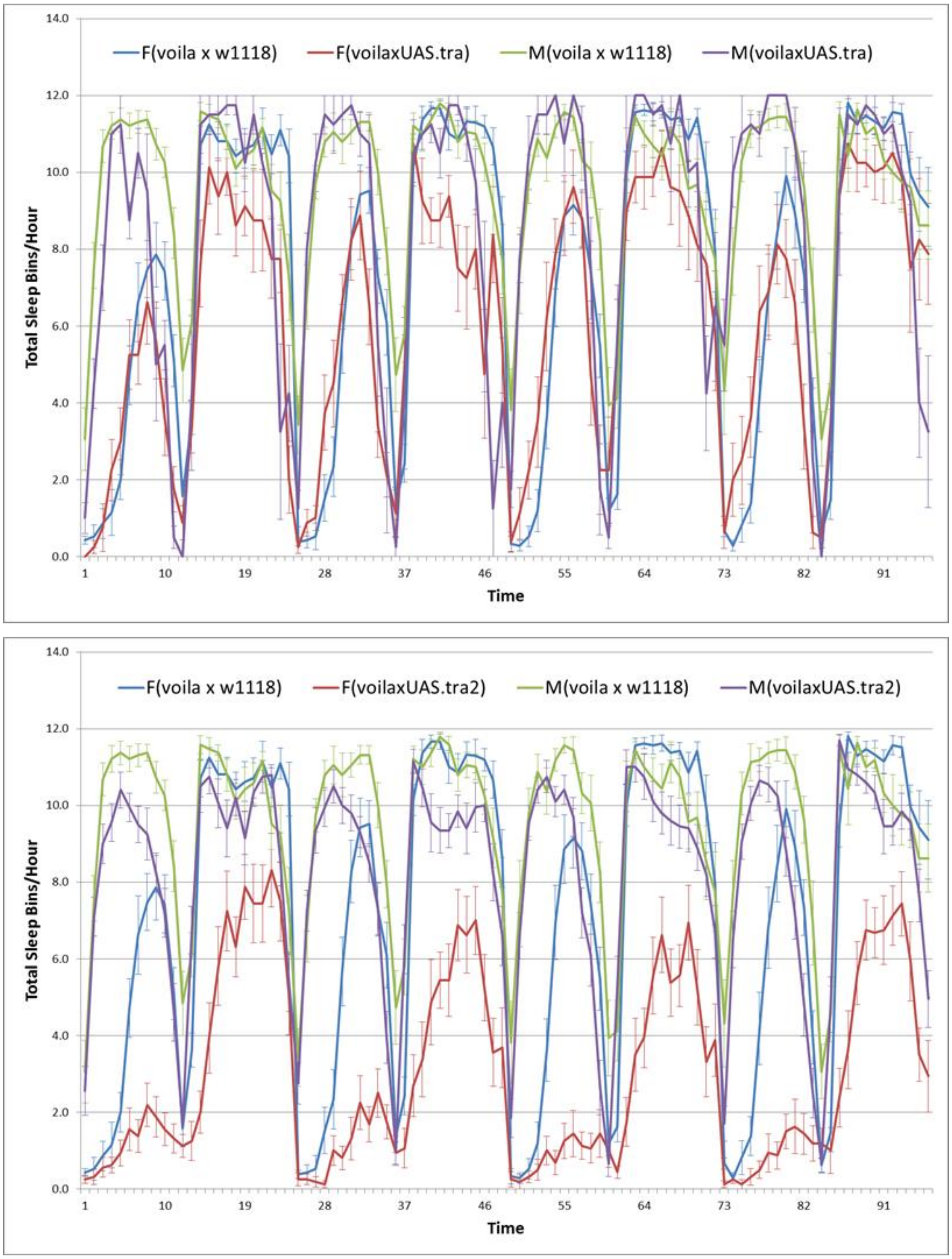

Figure S5. Sleep profiles in flies carrying the *voila*-GAL4 driver. The average total sleep (# 5 min bins/hour) over four days is depicted (n= > 20 for all genotypes). UAS-*tra* (feminsation of males) genotype (**top**) and UAS –*tra2* (masculinisation of females) genotype (**bottom**) of males females (M and F respectively). Error bars represent SE.

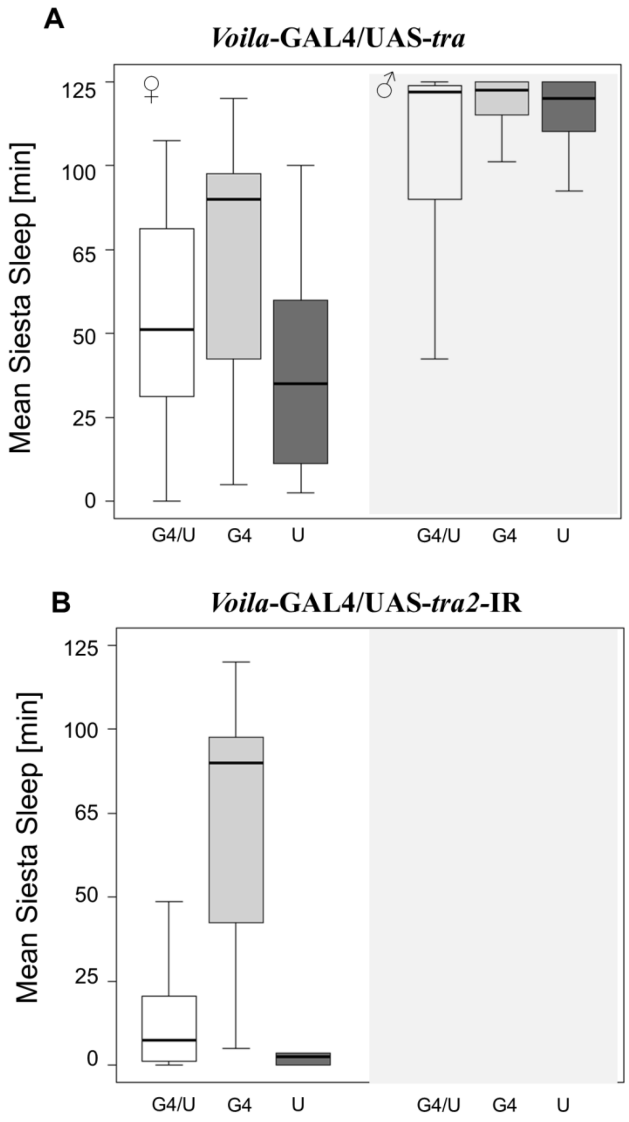

Figure S6. Siesta sleep following feminsation and masculinisation using the *Voila*-GAL4 driver. Box-plots showing siesta sleep in flies carrying voila-GAL4 driving (**A**) UAS-*tra* (feminsation of males), and (**B**) UAS –*tra2* (masculinisation of females). In each panel, the three left boxes show sleep in females, and the three right boxes (shaded grey) are for the males. The data shown in each panel represent siesta sleep for the GAL4/UAS genotypes (white, n= > 20 for all GAL4 lines; males and females) and the single transgene control genotypes (GAL4/+; light grey, UAS/+; dark grey) for both sexes. The line within each box represents the median siesta sleep averaged over 4 days (in minutes), and the boxes extend to 25 and 75 percentiles. No significant differences were present in males in the feminisation experiments, or among the females in the masculinisation experiments.
